# Patient Engagement With the Myderma Platform for Psoriasis During the COVID-19 Pandemic

**DOI:** 10.2196/39451

**Published:** 2023-04-19

**Authors:** Konstantinos Sfaelos, Stathis Kontodimas, Theodora Charisiadi, Nagia Chantzara, George Pesiridis, Eleftheria Tampouratzi

**Affiliations:** 1 LEO Pharmaceutical Hellas Athens Greece; 2 CORONIS Research SA Athens Greece; 3 Dermatological Department Tzaneio General Hospital Piraeus Greece

**Keywords:** psoriasis, social media, online health-related information, COVID-19, disease awareness, disease awareness website, digital campaigns, patient activation, patient engagement, COVID-19 pandemic

Social media’s impact on health care ranges from enabling the discovery of new medical knowledge and information to providing cost-effective ways to improve physician-patient communication [1]. This paper assessed social media’s usefulness in the psoriasis setting by analyzing the Myderma website, with a focus on the impact of the COVID-19 pandemic on users’ reachability and engagement.

LEO Pharmaceutical Hellas developed the Myderma website [2] (launched on September 1, 2016) to play an active role in supporting patients with psoriasis and their symptoms. The website provides access to a wide variety of topics, including disease-related information, available treatment options, and useful tips on psoriasis and improving quality of life. Myderma is also available through Facebook (launched on July 5, 2016) and YouTube (launched on May 31, 2016). This health care social media platform provides an interactive way to communicate, as users can participate in questionnaires and short polls about psoriasis and the ways it is affecting their lives. Patients with psoriasis are often stigmatized due to the high visibility of the disease; therefore, searching for information over the internet and taking advantage of the anonymity provided might be beneficial [3,4]. Through Myderma, patients could also look for a nearby dermatologist, simply by clicking on the “Find Dermatologist” icon, which leads to the Hellenic Society of Dermatology and Venereology, with call-to-action requests.

Participants in our analysis included visitors to the Myderma website as well as its Facebook, Instagram (launched in October 2019), and YouTube pages. Two periods were defined: before the COVID-19 pandemic, from January to December 2019, and during the COVID-19 pandemic, from January 2020 to June 2021. There were no major differences in the company’s advertising expenditure during the two periods.

During the COVID-19 pandemic, a significant increase was observed in the number of visitors to the website, from 35,067 users prior to the pandemic to 82,479 users during the pandemic. The increase was consistent across all age groups (Tables 1 and 2).

Despite significant progress over the last decades, psoriasis is still associated with increased stigmatization, accompanied by significant psychosocial burden, especially for women [4]. This was reflected by the increased prevalence of female users of the website and Facebook.

The COVID-19 pandemic led to increased psychosocial burden, changes in treatment, and disrupted access to dermatologists [5]. During COVID-19, lockdown measures were implemented for approximately 7 months in Greece [6]. Polls and questionnaires conducted on the website or via Facebook and Instagram during the pandemic confirmed that most participants reported no access to a treating physician for over a year. Surprisingly, despite the disrupted relationship with their physicians and associated undertreatment, there was no increase in visits related to psoriasis treatment or COVID-19 vaccines.

Overall, our findings reveal that health care social media capabilities play an important role in patient engagement in challenging disease state settings, including in the psoriasis setting. Most importantly, health care–related social media platforms perform well during periods when the regular patient-physician relationship has been disrupted, such as during the COVID-19 pandemic.

**Table 1:** Distribution of participants who visited the Myderma website and social media pages by age group and gender.

Characteristic			Website, n (%)		Facebook, n (%)		YouTube (%)^a^
**Age group (years)**							
	<18	—^b^		—		2.31	
	18-24	14,831 (8.22)		1770 (4.00)		20.03	
	25-34	40,037 (21.83)		6551 (15.80)		19.38	
	35-44	40,141 (21.97)		8880 (21.30)		19.37	
	45-54	37,959 (20.85)		10,718 (26.00)		18.15	
	55-64	29,788 (16.28)		8604 (20.80)		13.17	
	≥65	19,873 (10.84)		4902 (12.10)		7.59	
**Gender**							
	Male	63,237 (35.27)		8666 (19.60)		49.23	
	Female	117,128 (64.73)		32,798 (80.40)		50.77	

^a^Counts were not provided by YouTube.

^b^Not applicable.

**Table 2:** Distribution of participants who accessed the website by age group.

Age group (years)	Jan 1, 2019, to Dec 31, 2019 (n=35,067), n (%)	Jan 1, 2020, to Jun 30, 2021 (n=82,479), n (%)	Jan 1, 2019, to Jun 30, 2021 (n=117,546), n (%)
18-24	1560 (4.45)	7500 (9.09)	9060 (7.71)
25-34	8880 (25.32)	13,974 (16.94)	22,854 (19.44)
35-44	7424 (21.17)	18,297 (22.18)	25,721 (21.88)
45-54	5939 (16.94)	21,161 (25.66)	27,100 (23.05)
55-64	6260 (17.85)	13,487 (16.35)	19,747 (16.80)
≥65	5004 (14.37)	8060 (9.77)	13,064 (11.11)
